# Homeotic transformation in a terrestrial isopod: insights into the appendage identity in crustaceans

**DOI:** 10.1007/s00114-023-01875-4

**Published:** 2023-09-19

**Authors:** Naoto Inui, Toru Miura

**Affiliations:** Misaki Marine Biological Station, School of Science, The University of Tokyo, Misaki, Miura, Kanagawa 238-0225 Japan

**Keywords:** Crustacean, Malformation, Pleopod, Pereopod, Serial homolog

## Abstract

In many crustacean species, an individual possesses both uniramous and biramous appendages that enable us to compare the two types on the same genetic background. Therefore, among the diverse morphologies of arthropod appendages, crustacean biramous appendages provide interesting subjects for studying the developmental mechanisms underlying appendage modifications. In this study, we report a malformed specimen of the terrestrial isopod *Porcellio scaber*, in which one of the pleopods was transformed into a different structure. Morphological observations of exoskeletons and musculatures by confocal scanning laser microscopy revealed that the transformed appendage was three-segmented, with at least the apical two segments having pereopod-like musculoskeletal structures. The apical segment of the transformed appendage lacked muscles, and the following segment had a pair of muscle bundles. These findings together with those of some previous studies of gene expression patterns in this species suggest that this anomaly could be caused by homeotic transformation of a flap-like pleopod into a three-segmented pereopod tip, which may be a homologous structure of the pleopod.

## Introduction

The diversified appendages of arthropods contribute to their success, and the relationships and homologies of their appendages have been controversially discussed (Boxshall 2004; Haug et al. 2012; Bruce 2021). Among arthropods, crustaceans show morphological and functional diversity of appendages in an individual as serial homologs (Haug et al. 2012; Martin et al. 2016). They allow us to study differences in developmental mechanisms between appendages and the evolutionary origins of differences between appendages, such as the relationships between biramous and uniramous appendages (Wolff and Scholtz 2008).

One approach to analyzing developmental mechanisms is teratology, which involves the study of malformed individuals from the natural environment and the developmental factors that contribute to their formation (Minelli and Munari 2013; Scholtz 2020). Although recent advances in molecular developmental biology are reducing the importance of descriptive teratology, the knowledge gained from malformed individuals remains valuable, especially in non-model species that are difficult to manipulate experimentally, or when discussing the underlying developmental mechanisms that cause malformations (Minelli and Munari 2013; Akkari et al. 2014; Scholtz and Brenneis 2016; Di et al. 2018).

Numerous malformed individuals have been reported in crustaceans. These were caused mainly by developmental errors, disfunction of hormonal organs, or parasite infestations (Scholtz 2020). However, in contrast to hexapods and chelicerates, fewer cases have been reported in crustaceans, and most of these reports have been in decapods (Scholtz 2020). There are only a few examples of other malacostracans, such as isopods and amphipods (Scholtz 2020).

Here, we report a specimen of the terrestrial isopod *Porcellio scaber* (Oniscidea: Porcellionidae) with a notable malformation of the pleopod. Based on morphological examination, the developmental mechanisms suggested by the phenotype are discussed in comparison with the findings of previous studies.

## Materials and methods

### Animals

**Fig. 1:**
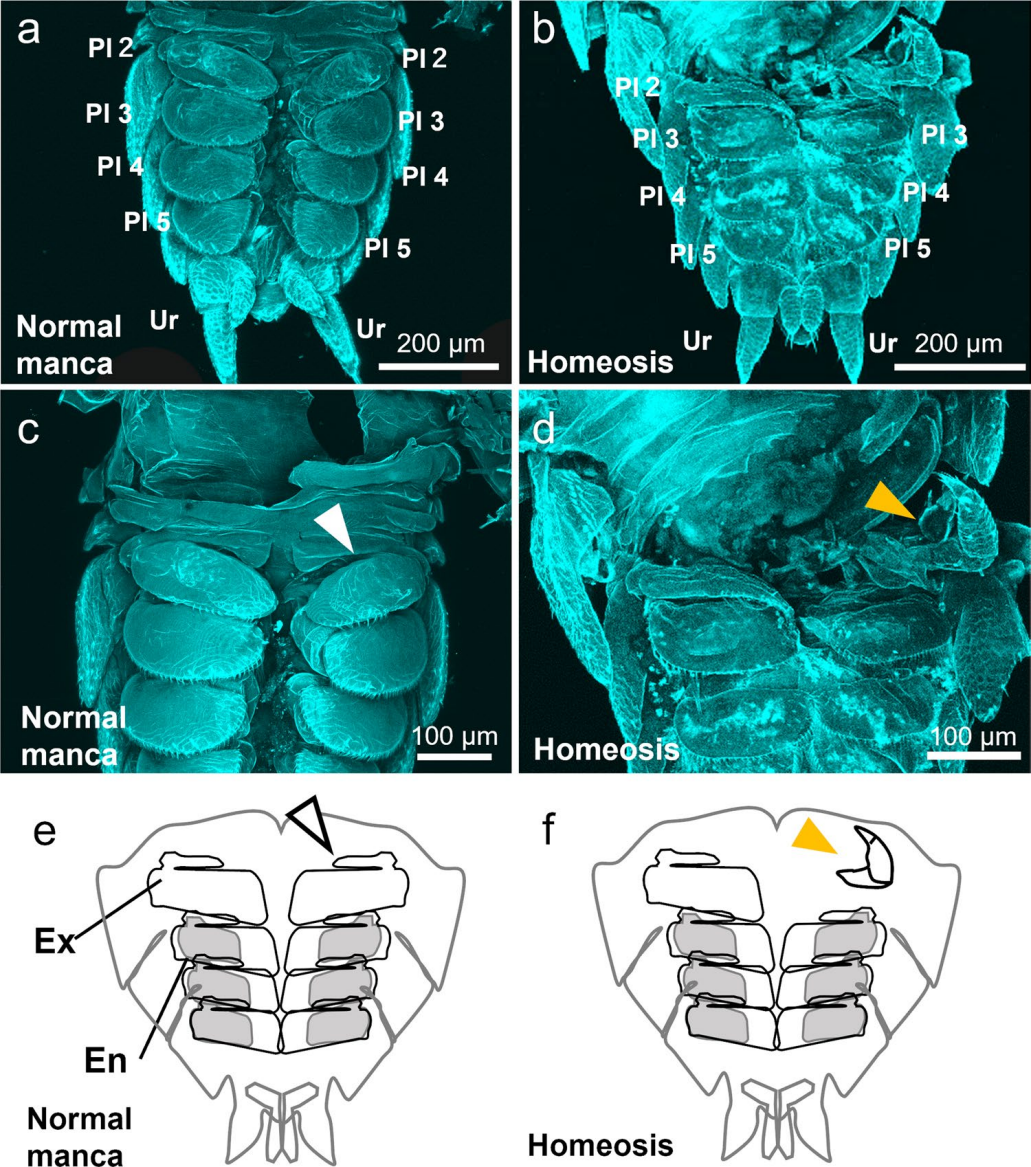
Homeotic transformation of *P. scaber*. Normal (**a**, **c**, **e**) and malformed (**b**, **d**, **f**) mancae were observed by detecting cuticle autofluorescence in confocal stack images. The fifth and sixth pereopods were removed for observation. **a**, **b** Ventral view of abdomen. **c**, **d** Enlarged view of abdomen. **e**, **f** Schematic images of the abdomen shown in **c**, **d**. The white arrowheads (**c**, **e**) indicate normal pleopod 2, and the orange arrowheads (**d**, **f**) indicate the transformed structure. Abbreviations: Pl, pleopod; Ur, uropod; Ex, exopod; En, endopod

*Porcellio scaber* were collected from the soil on the Misaki Marine Biological Station campus and kept in plastic cases according to a previous study (Inui et al. 2022). One malformed juvenile was found in the marsupium of a collected female (Fig. 1). At least 100 ovigerous females have been observed, each with approximately 20 to 40 juveniles, and no other obvious malformed individuals were found. The developmental stage of the individual corresponded to the manca 1 stage, immediately after hatching (Inui et al. 2022). The malformed individual and normal mancae were removed from the marsupia of ovigerous females using tweezers and fixed in 4% paraformaldehyde (PFA) in 1× phosphate-buffered saline (PBS). They were then preserved in 0.3% Triton-X 100 in 1× phosphate-buffered saline (PBT) at 4 °C until use.

### Morphological observation by fluorescent staining

To examine their morphological characters, exoskeletons and musculature of the specimens were investigated by fluorescent staining and detection of cuticle autofluorescence. Pereopods and the transformed appendage were removed under a stereoscopic microscope before the preparation. The fixed samples were washed in PBT for 15 min at least three times before staining. Musculature (F-actin) was stained with rhodamine-phalloidin (1:40; Invitrogen, Paisley, UK), for 1 h at room temperature. Then, the stained samples were washed for 15 min in PBT at least three times, mounted on glass-bottom dishes, and observed using a confocal microscope (FV3000; Olympus, Tokyo, Japan) with a laser excitation of 405 nm (autofluorescence) and 561 nm (rhodamine-phalloidin). Images were processed with software FV31S-DT (Olympus, Tokyo, Japan) and GIMP-2.10 (https://www.gimp.org/).

## Results

The normal manca had four pairs of pleopods (Fig. 1a, c, e). In the malformed individual, the second pleopod on the right side was not identified (Fig. 1c, e; white arrowheads), and a different structure was observed (Fig. 1d, f; orange arrowheads). This structure contained a claw-like segment at the tip and was similar to the pereopods of normal individuals (Fig. 1d).

**Fig. 2:**
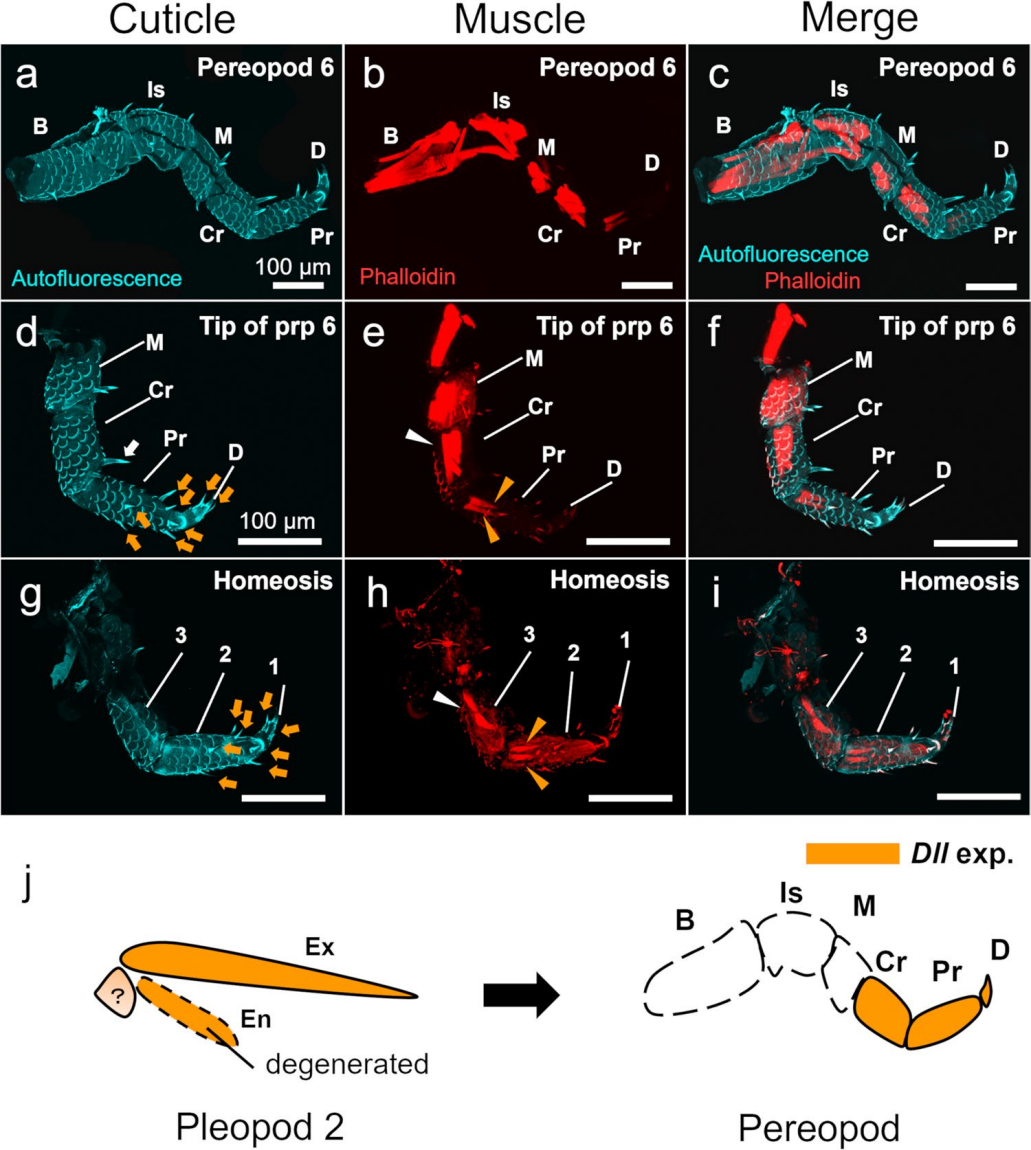
Musculoskeletal structures of the transformed appendage and interpretation. Cuticles (**a**, **d**, **g**), muscles (**b**, **e**, **h**), and merged structures (**c**, **f**, **i**) are shown in confocal images. **a**–**c** Normal sixth pereopod. **d**–**f** Tip of sixth pereopod. **g**–**i** Transformed appendage. Numbers indicate segments of structure (**g**–**i**). White arrow indicates a seta in carpus of sixth pereopod (**d**). Orange arrows indicate setae in propodus and dactyl of sixth pereopod (**d**) and last two segments of transformed appendage (**g**). White arrowheads indicate muscles in carpus of sixth pereopod (**e**) and third segments of transformed appendage (**h**). Orange arrowheads indicate a pair of muscle bundles in the second segments of the sixth pereopod (**e**) and the transformed appendage (**h**). **j** Schematic images of the transformed appendage. Orange segments indicate *distal-less* gene expression based on Abzhanov and Kaufman (2000b) and Hejnol and Scholtz (2004). Autofluorescence (blue), phalloidin (red). Abbreviations: B, basis; Is, ischium; M, merus; Cr, carpus; Pr, propodus; D, dactyl; Prp, pereopod; Ex, exopod; En, endopod

To investigate the morphology of the transformed appendage, the musculoskeletal structures were compared with those of the normal pereopod. The normal pereopod consisted of six segments (basis, ischium, merus, carpus, propodus, and dactyl) with muscles inside the exoskeleton (Fig. 2a–c). Each segment had setae, and well-developed musculature was observed in all segments except the dactyl (Fig. 2a–f). The transformed appendage consisted of three segments (Fig. 2g–i), with the exoskeletal structure and the position of the setae, especially on the two apical segments, corresponding well to the propodus and dactyl of the normal pereopod (Fig. 2d, g; orange arrowheads). In contrast, the third segment of the transformed appendage, although similar in morphology to the carpus of the pereopod, lacked distinct setae and did not correspond to the setae on the carpus (Fig. 2d; white arrow).

The apical-most segment of the transformed appendage lacked muscles, and the following segment had a pair of muscle bundles (Fig. 2h). This was similar to the dactyl and propodus of the normal pereopod (Fig. 2e, h; orange arrowheads). The muscles of the third segment of the transformed segment were also present in the same position as those of the pereopod carpus, although they were more reduced (Fig. 2e, h; white arrowheads).

## Discussion

The transformed appendage had a musculoskeletal structure similar to that of the tip of a normal pereopod (Fig. 2), suggesting that a homeotic transformation from a pleopod to a pereopod had occurred. In *P. scaber*, the endopod of the second pleopod develops during embryogenesis, but subsequently degenerates and remains as the vestigial bud at the manca stage (Wolff 2009; Milatovič et al. 2010). Since no exopod could be identified on the transformed appendage, it is likely that the exopod of the pleopod or the entire pleopod has been transformed into a pereopod. In a normal biramous appendage, where both exopod and endopod are present, transformation of the exopod to a pereopod (telopod) is extremely unlikely (Scholtz 2020). Given the presence of the vestigial endopod (Milatovič et al. 2010), it is therefore likely that the entire pleopod was transformed. Based on the number of individuals observed, the malformations observed here are considered to be extremely rare and are unlikely to be due to any environmental factors. As the manca 1 stage is the stage immediately after hatching, the malformation was not due to regeneration but to a developmental abnormality.

Similar cases of homeotic transformation have been reported in decapods, including the transformation of the entire pleopod or the endopod of the pleopod into a pereopod with reduced segments (Young 1933; Fausto-Filho and Costa 1977). The present observations suggest that the reduced segments correspond to the tip of the pereopods.

In model organisms such as fruit flies, the homology of arthropod appendages has been deeply discussed based on the expression patterns of developmental genes (Bruce 2021). The expression of these genes, including *extradenticle*, *dachshund*, and *distal-less*, during embryogenesis has also been described in *P. scaber.* (Abzhanov and Kaufman 2000b; Hejnol and Scholtz 2004). Given these known gene expression patterns, the morphology observed in the present study suggests that the pleopod has transformed into the apical three segments of the pereopod (dactylus, propodus, carpus) (Fig. 2J). Furthermore, a pleopod to pereopod transformation similar to the present case was observed when the *Abdominal-B* function was inhibited in the abdomen of the model amphipod *Parhyale hawaiensis* (Martin et al. 2016). In *P. scaber*, hox gene *Ultrabithorax* was expressed in the developing thorax and *abdominal-A* in the developing abdomen (Abzhanov and Kaufman 2000a). Therefore, it is possible that the loss of abdominal identity in only one side of the pleopod led to the pleopod transformation in the present individual. However, the phenotypes obtained in the *Parhyale* studies were slightly different from that in the present case. In addition to the possibility that the transformation occurred in some of the forming cells, the loss of the endopod during embryogenesis (Wolff 2009) could have been responsible for the present phenotype.

In the present study, we report a terrestrial isopod in which a homeotic transformation occured in the pleopod, which was transformed into a pereopod. The individual described here can complement functional analyses of gene functions and also suggests that a flap-like pleopod and a pereopod tip are homologous in crustaceans. Since recent advances in molecular studies enable us to analyze such gene functions, future molecular studies can be predicted to reproduce a similar phenotype, leading to a better understanding of appendage homologies in crustaceans.
